# Quantifying pine processionary moth defoliation in a pine-oak mixed forest using unmanned aerial systems and multispectral imagery

**DOI:** 10.1371/journal.pone.0213027

**Published:** 2019-03-19

**Authors:** Adrián Cardil, Kaori Otsu, Magda Pla, Carlos Alberto Silva, Lluis Brotons

**Affiliations:** 1 School of Agrifood and Forestry Science and Engineering, University of Lleida, Lleida, Spain; 2 Tecnosylva SL, Parque tecnológico León, León, Spain; 3 CREAF, Cerdanyola del Vallès, Spain; 4 InForest Joint Research Unit, (CTFC-CREAF) Solsona, Spain; 5 NASA Goddard Space Flight Center, Biospheric Sciences Lab, Greenbelt, Maryland, United States of America; 6 Department of Geographical Sciences, University of Maryland, College Park, Maryland, United States of America; 7 CSIC, Cerdanyola del Vallès, Spain; Western University, CANADA

## Abstract

Pine processionary moth (PPM) feeds on conifer foliage and periodically result in outbreaks leading to large scale defoliation, causing decreased tree growth, vitality and tree reproduction capacity. Multispectral high-resolution imagery acquired from a UAS platform was successfully used to assess pest tree damage at the tree level in a pine-oak mixed forest. We generated point clouds and multispectral orthomosaics from UAS through photogrammetric processes. These were used to automatically delineate individual tree crowns and calculate vegetation indices such as the normalized difference vegetation index (NDVI) and excess green index (ExG) to objectively quantify defoliation of trees previously identified. Overall, our research suggests that UAS imagery and its derived products enable robust estimation of tree crowns with acceptable accuracy and the assessment of tree defoliation by classifying trees along a gradient from completely defoliated to non-defoliated automatically with 81.8% overall accuracy. The promising results presented in this work should inspire further research and applications involving a combination of methods allowing the scaling up of the results on multispectral imagery by integrating satellite remote sensing information in the assessments over large spatial scales.

## 1. Introduction

The area covered by forest ecosystems in the Mediterranean has increased during the last century due to land abandonment and climate change impacts, which have led to significant changes in forest dynamics [1–3]. These forest changes an increase in the effects of pests on trees, partially due to more frequent large-scale outbreaks becoming an increasingly important disturbance in forest dynamics [4,5]. Amongst these pests we can highlight the increasing impact of the pine processionary moth (*Thaumetopoea pityocampa* Dennis and Schiff., Lepidoptera: Notodontidae; henceforth PPM), one of the main pests of Pinus sp., a native species of the Mediterranean region including North Africa, southern Europe and some areas of the Middle East [6]. Life cycle is characterized by a one-year development cycle for short-lived female moths which typically live for 1 or 2 days and longer-lived males [7]. The cycle involves adult emergence in summer (June–September), larval feeding during fall and winter, and pupation in soil followed by a short or prolonged diapause up to several years under specific circumstances [4,7–9]. The area affected by PPM in Europe is expanding northwards to higher latitudes and upwards to higher altitudes from where it was absent, probably as a result of increasing winter temperatures [6].

PPM caterpillars feed on conifer needles resulting in a general weakening of trees and eventually cause large scale defoliation at the stand and landscape levels. Intense defoliation negatively affects height and radial tree growth, increase the mortality rate of saplings and reduce the reproduction capacity of trees [10–12]. At the same time, PPM can trigger a decrease in tree resistance and resilience against other disturbances such as forest fires, drought conditions or other pests [5,13,14] that could influence and modify the tree species composition in mixed forests. PPM damages are associated with the spatial and temporal complexity of Mediterranean landscapes, as well as with its life cycle. Regarding the spatial complexity, trees in fragmented small stands suffer PPM damage with varying intensities. However, the current pattern of outbreaks at landscape level is still largely unknown [15]. Regarding the temporal complexity, PPM defoliation is maximum at the beginning of spring. During the spring and summer, if weather conditions are not extremely stressful, the vegetation generally recovers its initial greenness. There is also lack of knowledge about the effects of forest diversity and mixed versus monospecific forests on overall pest tree damage [16]. Several studies showed that diverse forests are less prone to pest insects than monoculture forests [17], which suggests associational resistance [18]. However, recent studies also reported more damage in mixed forests suggesting associational susceptibility [19], or simply no effect of diversity [20]. However, recovery time appears to depend on the site characteristics of each forest [21]. Thus, quantifying defoliation processes at landscape level is key to improve the knowledge of PPM damage patterns and consequently to better guide control measures or improve ecological conditions to increase forest resistance and resilience to this kind of damages.

Historically, the degree of infestation and mortality by PPM in pine stands [9,15,22] has been visually assessed in the field [10] by forestry technicians or through interpretation of aerial photographs [23]. However, field inventories are expensive and require a large amount of manpower and resources while aerial photographs taken in periods relevant to assess PPM impacts are not always readily available or may lack sufficient temporal details. In addition, visual interpretation can be subjective if not carefully validated and may not always provide the required quantitative information when assessing spatial variability in PPM impacts. More recently, multi-temporal satellite images and remote sensing products such as Landsat Thematic Mapper (TM), Moderate Resolution Imaging Spectroradiometer (MODIS), TerraSAR-X and light detection and ranging (LiDAR), have been extensively used in monitoring forest health attacks by pest insects [23–26] and found to be useful for insect outbreak surveys at large scale [27]. Yet, even these medium-resolution remote sensing products have been unable to capture the spatial heterogeneity in agroforestry mosaics or complex defoliation patterns caused by PPM at local scale [27] and, especially, in mixed forests at tree level.

Currently, unmanned aerial systems (UAS) platform have also become suitable tools to perform small-scale analyses as well as local-scale sampling, assessment and validation that can be complemented and integrated with aerial or satellite imagery for broader spatial scale analyses [28,29]. Most recent years have experienced an enormous increase in the use of UAS due to low infrastructure requirements, ease of deployment, acquisition and suitability for photogrammetric workflows [30,31]. The current availability of photogrammetric software allows for handling and processing of large spatial datasets. UAS have been applied in fine-scale studies to inventory forest resources [30], map diseases [32], assess pest damages at tree level [5,27,33,34] and at the landscape level [35,36], quantify spatial gaps or estimate post-harvest soil displacement [37]. UAS platforms are establishing a niche in low cost image acquisition at local scales that make this technology an alternative cost-effective option in forestry applications. However, most common cameras employed in UAS surveys are digital RGB cameras [38], or are adapting one of the visible bands for NIR imagery acquisition [30]. To date, only a few studies with multispectral cameras such as Tetracam ADC Lite, MicaSenseRedEdge [39] or SEQUOIA (Parrot SA, Paris, France) [40] have demonstrated improvements in the analysis of fine-scale forest dynamics.

In this study, we aimed at evaluating the potential use of multispectral high-resolution imagery acquired from a UAS platform and image processing techniques to quantitatively assess PPM impact on a pine-oak mixed forest. To achieve this objective, we used point clouds and multispectral orthomosaics from UAS generated through photogrammetric processes. Point clouds have proven useful to generate high resolution digital surface models (DSM) from which it is possible to automatically identify and delineate individual trees [28,30,41]. We hypothesized that multispectral information will enable us to calculate vegetation indices such as NDVI to objectively quantify the degree of defoliation of the pines previously delineated from DSM [21,42,43] after identifying tree species [44,45]. Field data was used to validate our results and assess the level of detail and accuracy of our method to investigate the spatial dynamics of the pest. More precisely, the presented study attempts to develop a method using high resolution multispectral images collected with UAS to automatically quantify defoliation by PPM for individual trees and classify them as non-defoliated, partially defoliated and completely defoliated in a pine-oak mixed forest.

## 2. Materials and methods

### 2.1 Study area

**Fig 1:**
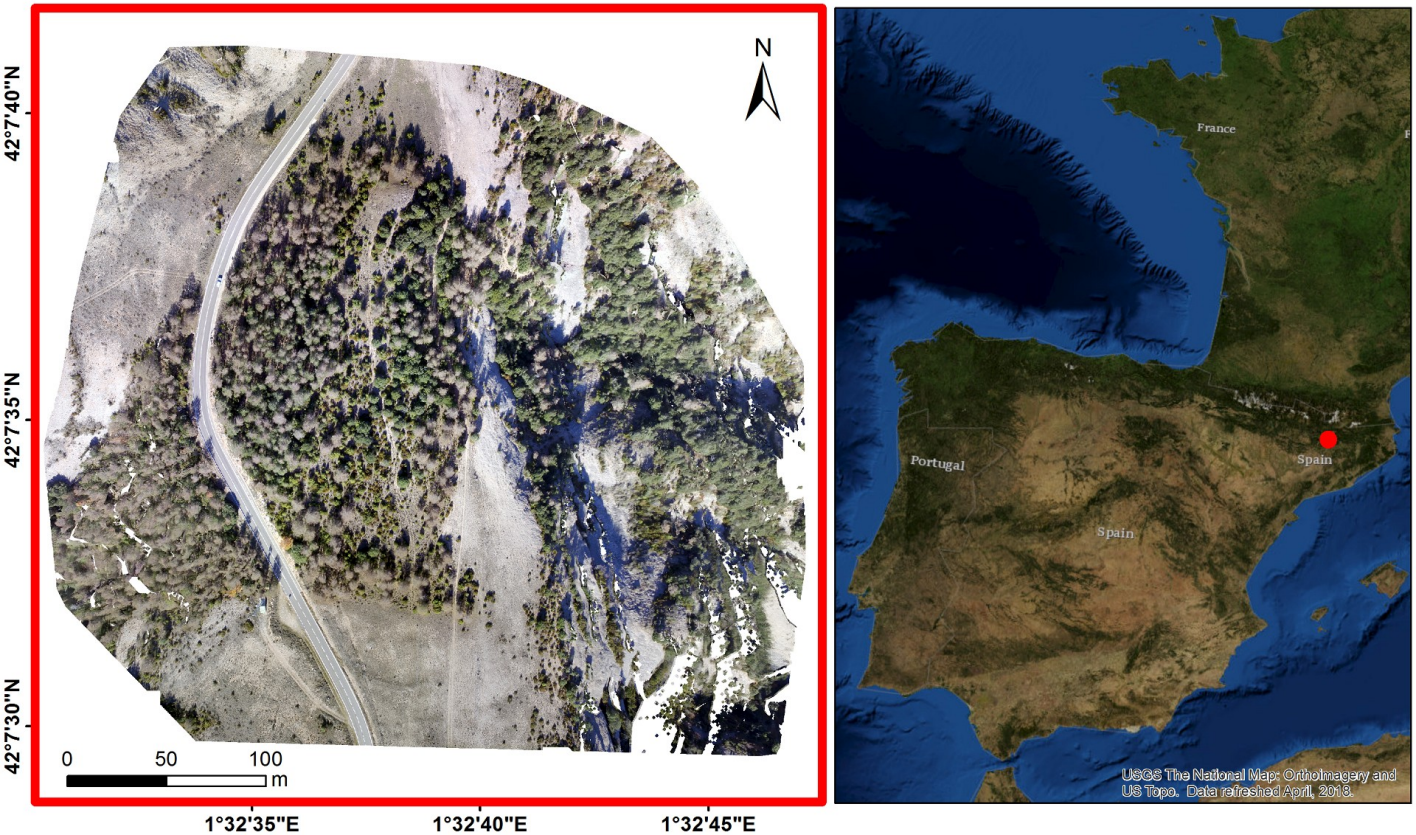
An RGB orthomosaic of the study are, Codo in Catalonia (Spain). Base map source: USGS National Map Viewer.

The study was conducted in “Codo” site (longitude = 1.544°; latitude = 42.127°; altitude = 1300m), a mixed forest area located near the city of Solsona (Lleida, Catalonia, NE Spain, Fig 1) dominated by holm oaks (*Quercus ilex* L.) and Scots pines (*Pinus sylvestris* L). The dimension of the study area was 350 × 350m (12.25 ha) representing a Mediterranean climate zone characterized by hot dry summers and mild wet winters.

### 2.2 UAS-based image acquisitionand data preprocessing

Multispectral high spatial resolution image data was collected using a quadcopter (UAS Phantom 3 from DJI) in two successive flights with one hour gap between both. This UAS is capable of autonomous, waypoint flight following a preplanned route. In the first flight, the UAS was equipped with the multispectral camera Parrot SEQUOIA and, in the second with RGB PC300S Phantom 3 camera, to create RGB orthomosaics. The Parrot SEQUOIA camera has four 1.2-megapixel monochrome sensors that collect global shutter imagery along four discrete spectral bands: green (center wavelength -CW-: 550 nm; bandwidth -BW-: 40 nm), red (CW:660 nm; BW: 40 nm), red edge (CW: 735 nm; BW: 10 nm) and near infrared -NIR- (CW: 790 nm; BW: 40 nm). The horizontal (HFOV), vertical (VFOV) and diagonal (DFOV) fields of view of the multispectral camera are 70.6°, 52.6° and 89.6°, respectively, with a focal length of 4 mm. With a flight altitude of 120 m and an image overlap of 80%, a ground sample distance (GSD) of 15 cm was achieved. The camera was bundled with an irradiance sensor to record light conditions in the same spectral bands as the multispectral sensor. The total weight of the multispectral camera with the irradiance sensor is 107 g. This camera stored 16-bit RAW files (based on 10-bit data) during image shooting. The ISO value and exposure time were set to be automatic. Every image capture setting was saved in a text metadata file together with the irradiance sensor data. The text metadata files register information about ISO, aperture, shutter speed, sensor response and optical system and vignetting. The optical system and vignetting are registered by the irradiance sensor. Additionally, irradiance panels available by AIRINOV photos were taken at the beginning of the flight. An absolute reference for each spectral band was set through the calibration target, which allow getting absolute reflectance values. The aerial survey was carried out on November 26^th^, 2017 within one hour of the solar zenith in a clear sky and no winds. Finally, a total of 202 SEQUOIA photographs were collected to cover the study area of Codo.

Pix4Dmapper desktop photogrammetric software (https://pix4d.com/; accessed on 2 April 2018) following the "Ag Multispectral" template was used to generate point clouds, 3D reconstruction, radiometric calibrations and correction and finally the orthomosaics. This software integrates computes vision techniques with photogrammetry algorithms [30] to obtain high accuracy in aerial imagery processing. Pix4Dmapper Pro computes key points on the single images and uses them to find matches between images. From these initial matches, the software runs several automatic aerial triangulation (AAT), bundle block adjustments (BBA) and camera self-calibration steps iteratively until optimal reconstruction is achieved [40]. Then, a densified point cloud is generated to obtain a highly detailed digital surface model (DSM) that will be used to generate the final reflectance orthomosaics maps for every plot. The reflectance maps were achieved applying radiometric calibrations and corrections. First, the images of the irradiance panels taken at the beginning of the flight allow the radiometric calibration. Second, we also applied a "Camera and Sun irradiance" radiometric correction to correct for factors that distort the true reflectance pixel values and achieving a radiometric trustful measure of the terrain reflectance taking into account the information registered in the text metadata files (EXIF and XMP tags) for every single photogram. Pix4Dmapper applies this calibration and correction process to every single photogram just before achieve the final reflectance orthomosaic for every spectral band. Once the radiometric corrections were done for every photogram, the final orthomosaic was generated through automated workflows and SFM (structure from motion) methods [46] with image identification and feature matching. After the initial alignment through bundle adjustment, the resultant sparse cloud was assessed for projection errors, followed by reconstruction of dense point clouds using the cartographic UTM projection system. With original photos projected onto the 3D models, blending the overlap areas produced the reflectance orthomosaic for each spectral band.

**Fig 2:**
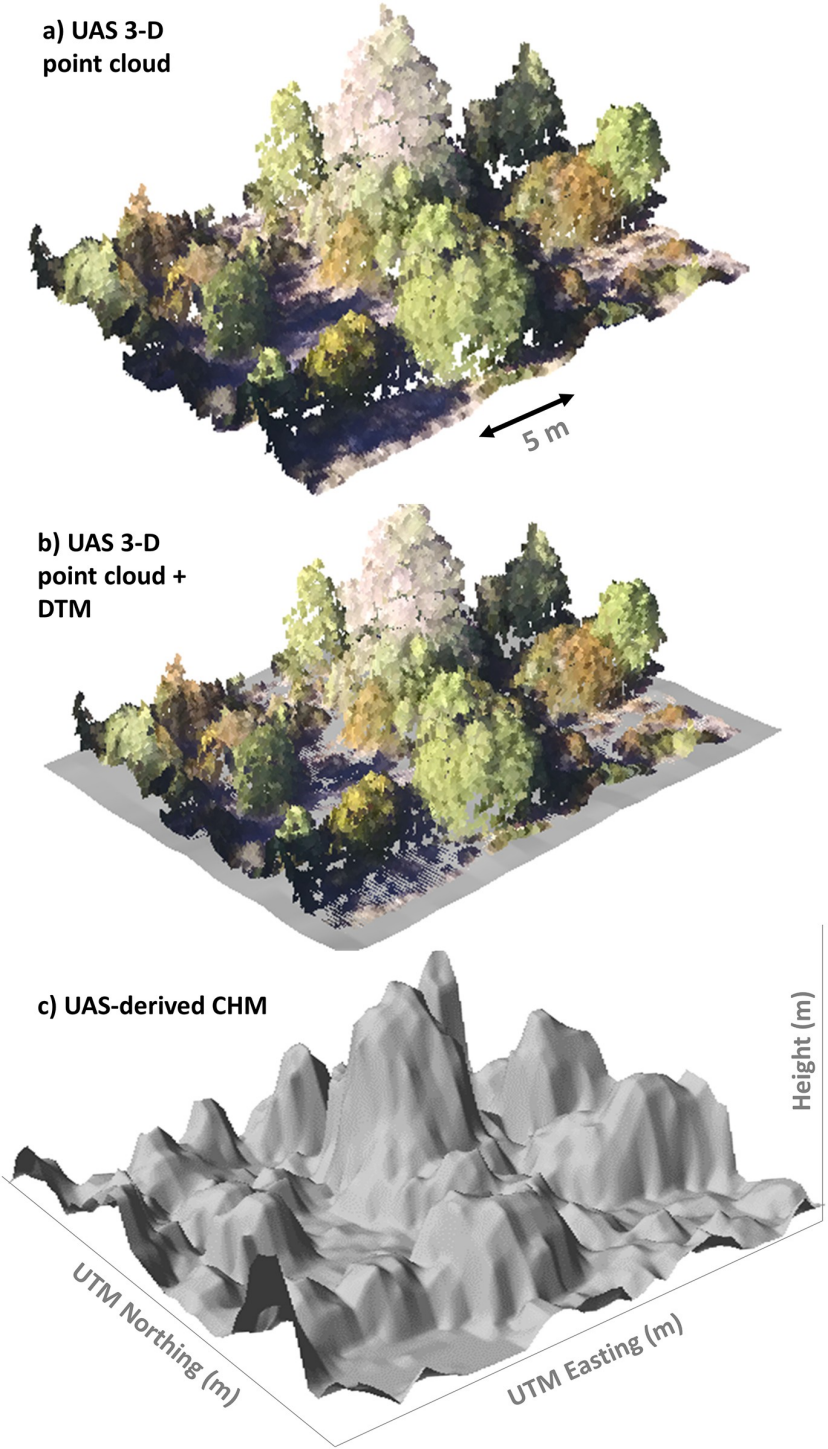
A canopy height model (CHM) derived from UAS-derived 3-D point cloud and a digital terrain model (DTM). (A) UAS-derived 3-D point cloud. (B) Digital terrain model. (C) Canopy height model.

The UAS-derived 3-D point cloud was used to compute a digital terrain model (DTM) and a canopy height model (CHM) based on the approach developed by Mohan et al. (2017) (Fig 2). First, one m DTM was created using the GridSurfaceCreate function in FUSION/LDV 3.42 [47] after classifying ground points using a progressive Triangulated Irregular Network (TIN) densification algorithm implemented in lasground (settings: step is 10 m, bulge is 0.5 m, spike is 1 m, offset is 0.05 m), LAStools [48]. Secondly, the UAS-derived 3-D point cloud was normalized to height above ground by subtraction of the DTM elevation from the Z coordinate of each point projected on the ground using the ClipData tool. Lastly, the CanopyModel function, also in FUSION/LDV, was used to compute the CHM at 0.5 m spatial resolution for the study site.

### 2.3 Field validation data

**Fig 3:**
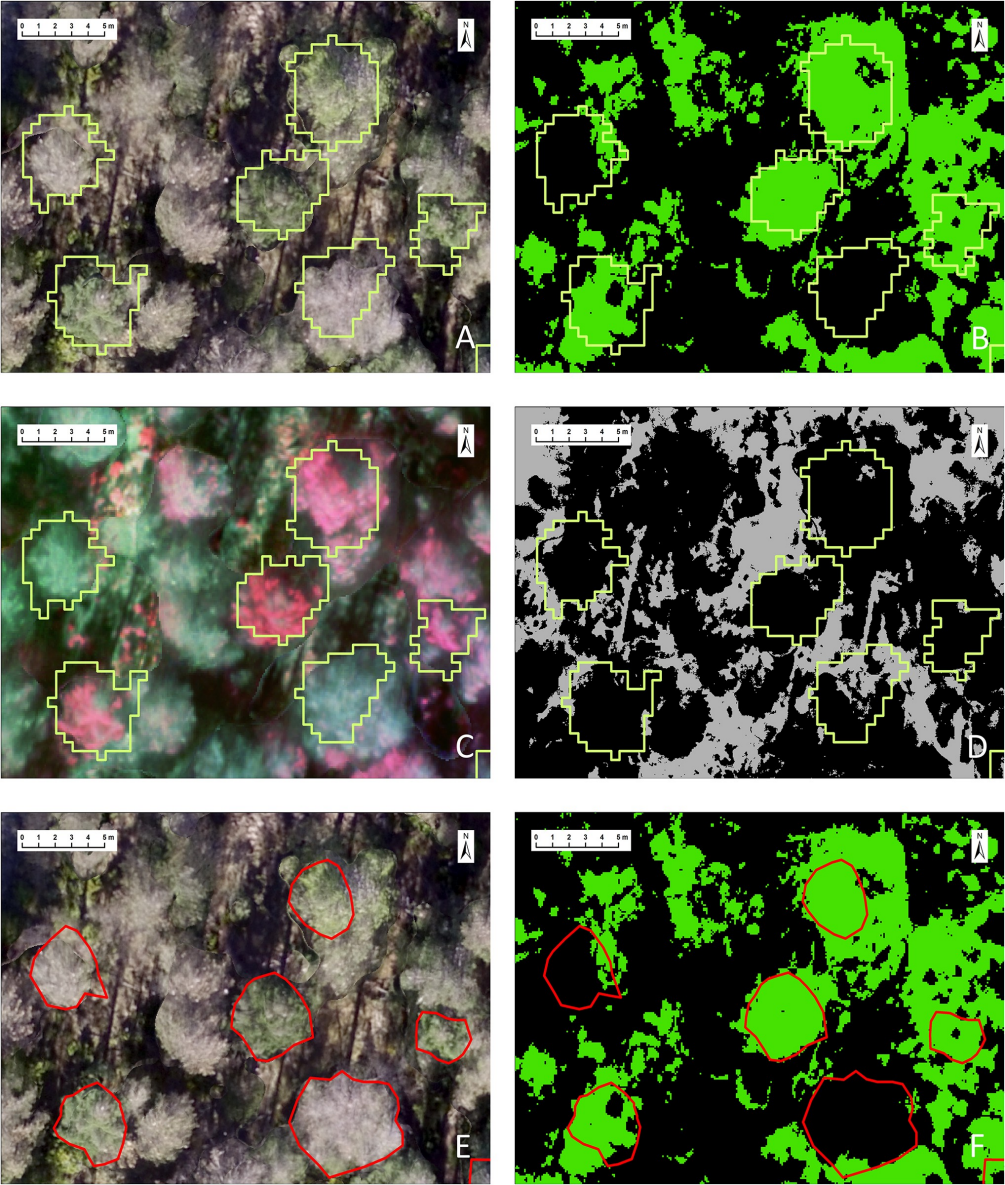
Object-based image analysis. (A) RGB orthomosaic image with crown surfaces delineated by ITDe. (B) Image classification with NDVI for non-defoliated (in green) surfaces and defoliated / non-vegetation background (in black) with ITDe. (C) Colour-infrared composite using green, red and nir reflectance bands with crown surfaces delineated by ITDe. (D) Unsupervised classification with ExG for isolating shaded pixels (in grey). (E) Manual digitization of tree crowns (MCDe) for validating the results by ITDe. (F) Image classification with NDVI for non-defoliated (in green) surfaces and defoliated / non-vegetation background (in black) with MCDe.

A randomly selected sample of 110 trees from the Codo forest was acquired through a visual assessment at tree level (Fig 3). The sample consisted of 25 holm oak (they were excluded from the analysis to assess percentage of defoliation at tree level because they were not defoliated), 13 non-defoliated pines, 29 partially defoliated pines and 43 completely defoliated pines depending on the level of defoliation. Pines were classified as non-defoliated, partially defoliated and completely defoliated trees when having defoliation < 15%, between 15% and 85%, and > 85%, respectively.

### 2.4 Data analysis

#### 2.4.1 Individual tree identification and delineation (ITDe)

The rLiDAR package [49] in R [50]was used in this study for individual tree delineation and defoliation assessment on the UAS-derived CHM. First, the FindTreesCHM function from this package, based on a local maximum algorithm, was applied for automatic detection of tree tops on the UAS-derived CHM using fixed tree and smoothed window sizes of 3x3 pixels. Secondly, the ForestCAS function from the same package, based on the Voronoi tessellation [51], was applied for individual tree delineation (ITDe) on the UAS-derived (Fig 1) based on Silva et al., (2017).

Independently, the 110 randomly selected trees in the field were manually onscreen-digitized on the orthomosaic image in order to assess the accuracy of the automatic ITDe and compare the results obtained with this method in terms of PPM defoliation. We analyzed spatial discrepancies in each tree between ITDe and manual tree crown delineation (MCDe) by using the Sørensen’s coefficient (SC; (Legendre and Legendre, 1998)) calculated as follows:

$$SC=\frac{2A}{2A+B+C}$$ (1)

where A is the area coded as “tree crown” for both ITDe and MCDe, B is the area coded as “tree crown” in the ITDe and “no crown” in the MCDe and, C is the area coded as “tree crown” in the MCDe and “no crown” in the ITDe. SC coefficient values range between 0 and 1, with values close to 1 indicating very high spatial agreement between the variables.

#### 2.4.2 Tree species identification and defoliation assessment based on NIR and RGB imagery

In addition to visible bands in RGB images, the near-infrared (NIR) reflectance of the multispectral camera was use for tree species identification and defoliation detection as a stress indicator, which may measure plant health more precisely than visibly evident greenness [23,43,52]. Thus, we considered normalized difference vegetation index (NDVI), a ratio between the red (R) and NIR values, as potentially the most robust and widely tested indicator in order to predict the tree species and defoliation degree [23].

$$NDVI=\frac{NIR-R}{NIR+R}$$ (2)

In order to separate the vegetation from the background to assess PPM defoliation, excess green index (ExG) was calculated in this study. Previous studies successfully applied this index together with NDVI to image classification in their object-based image analysis (OBIA)[53,54].

$$ExG=2[\frac{G}{R+G+B}]-\frac{R}{R+G+B}-\frac{B}{R+G+B}$$ (3)

After automatically identifying and delineating tree crowns of the 110 selected trees, we identified tree species by using the mean NDVI of pixel values for each of the delineated crown. The default threshold value to classify the selected trees as holm oak (NDVI ≥ 0.42) or pine (NDVI < 0.42) was selected by sensitivity analysis on the NDVI range in comparison with field data (increasing by 0.01) considering the mean NDVI of pixel values per each delineated crown.

Image classification with NDVI was first processed for separating defoliated crown surfaces from non-defoliated ones by threshold analysis. The default threshold value was selected by automated unsupervised classification and compared to sensitivity analysis on the NDVI range (increasing by 0.01) against the orthomosaic images. Then threshold classification was applied at the pixel level: defoliated in black (NDVI <0.27) or non-defoliated in green (NDVI ≥ 0.27) as illustrated in Fig 3B.

It was noted that some shaded pixels in the RGB orthomosaic image, corresponding to dark grey-black areas by heavy shadowing in Fig 3A, were not visually distinguishable between defoliated and non-defoliated crown surfaces. Therefore, we decided to exclude those from thresholding with NDVI for the purpose of effectively validating classification accuracy. Since NDVI in general can detect non-defoliated leaves in shaded pixels [53], it was not successful to achieve the threshold value of NDVI for isolating shaded pixels exclusively. However, calculating such specific vegetation index as ExG enabled automated isolation of shaded pixels by unsupervised classification, where ExG is lower than -0.06 (Fig 3D). Finally, we applied another class ‘shaded’ to automatically mask all shaded pixels, regardless of the NDVI value, so that those pixels were excluded from the crown surface area for further analysis.

To evaluate the accuracy of threshold classification (defoliated, non-defoliated, shaded), we show a confusion matrix by assessing 100 pixels that were randomly selected and visually interpreted against the orthomosaic image. The overall classification accuracy between classified imagery and reference imagery was calculate as:

$$Accuracy(\%)=\frac{number\;of\;pixels\;correctly\;classified}{total\;number\;of\;pixels\;referenced}$$ (4)

We systematically quantified the percentage of defoliation by PPM in each pine by using both the automatic ITDe and the MCDe (Fig 3B and 3F). For evaluating the overall defoliation degree at tree level, pixels classified as defoliated were grouped by tree ID and calculated as follows:

$$Defoliation\;per\;tree(\%)=\frac{number\;of\;defoliated\;pixels}{sum\;of\;defoliated\;and\;non-defoliated\;pixels}$$ (5)

We statistically analyzed relationships between the predicted defoliation degree with NDVI (X) and the observed defoliation % on the orthomosaic image (Y). The relationship is expressed in linear regression:

$$Y_{i}=aX_{i}+b$$ (6)

where Y*i* represents the percentage of defoliation per tree interpreted on the orthomosaic image, X*i* represents the portion of pixels classified as defoliated with NDVI per tree, and *a* and *b* are the slope and intercept of the regression line. Based on the same 110 selected sample trees from the study area used to MCDe, linear regression models were fitted to evaluate both the slope of the regression line and coefficient of determination (R^2^).

#### 2.4.3. Accuracy of defoliated tree identification and validation of percent defoliation

To assess image classification accuracy in our PPM defoliation estimates at tree level and to distinguish between non-defoliated, partially defoliated and completely defoliated trees, we computed a confusion matrix of the 110 selected trees that were field identified and compared them with the obtained image classifications. Validation of the defoliation classification was performed by comparing field measured defoliation against the classification derived defoliation at tree level using both ITDe (Fig 3A and 3B) and MCDe (Fig 3E and 3F).

## 3. Results

### 3.1 Accuracy of the individual tree detection and species identification

**Fig 4:**
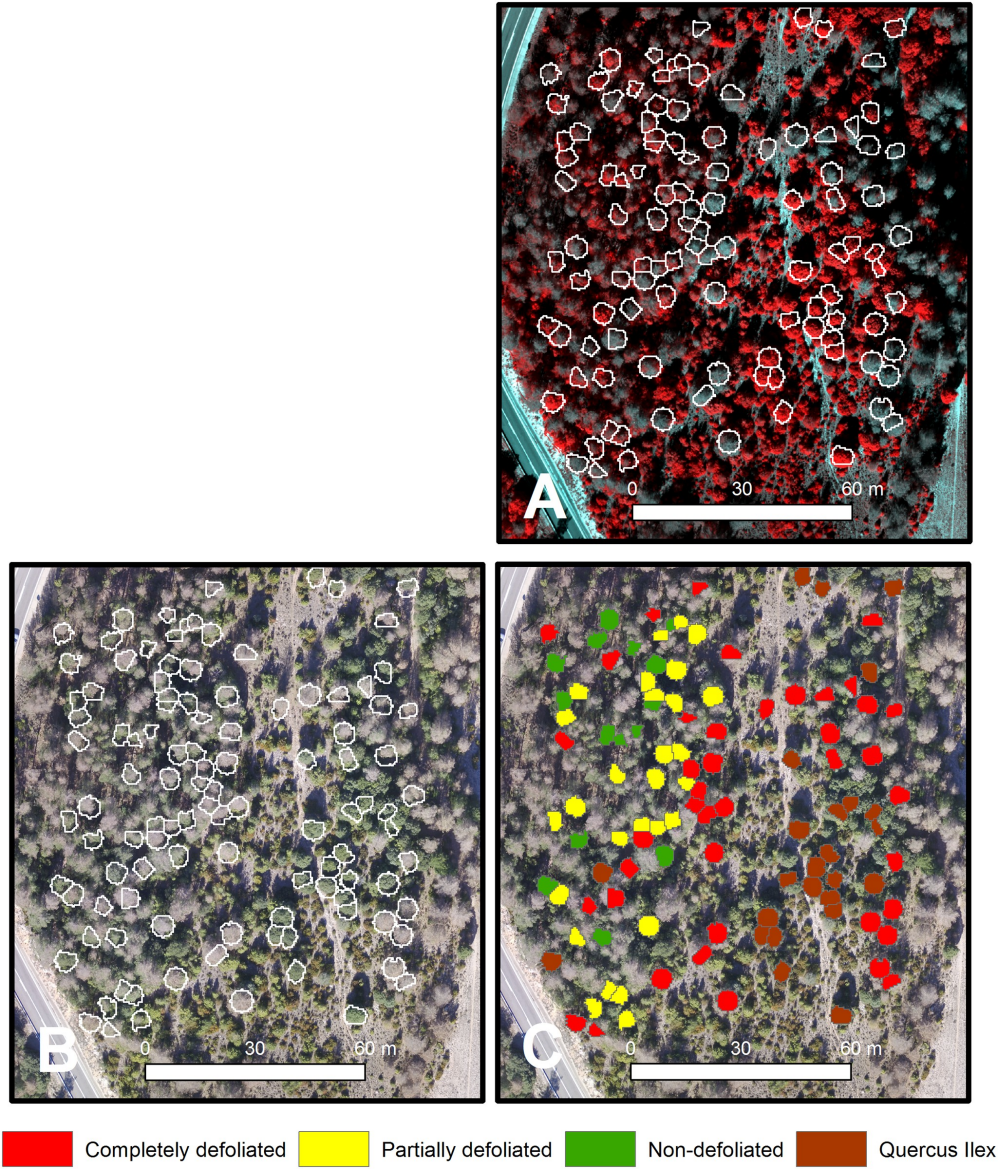
Tree species identification and pine processionary moth defoliation in pines in the Codo forest site. (A) Individutal automatic tree delination on the colour-infrared composite orthomosaic using green, red and nir reflectance bands. (B) Individutal automatic tree delination on the RGB orthomosaic. (C) Automatic tree classification in the field as holm oak or non-defoliated, partially defoliated and completely defoliated pine trhough multispectral high-resolution imagery.

The algorithm on the UAS-derived CHM effectively detected individual trees. The algorithm correctly identified all the 110 trees randomly selected through the field survey (Fig 4A). The accuracy of the automatic ITDe was also analyzed in comparison to the MCDe on the orthomosaic. The overall spatial agreement of crown area for the 110 selected trees was high with a mean SC of 0.75 and a standard deviation of 0.11.

**Fig 5:**
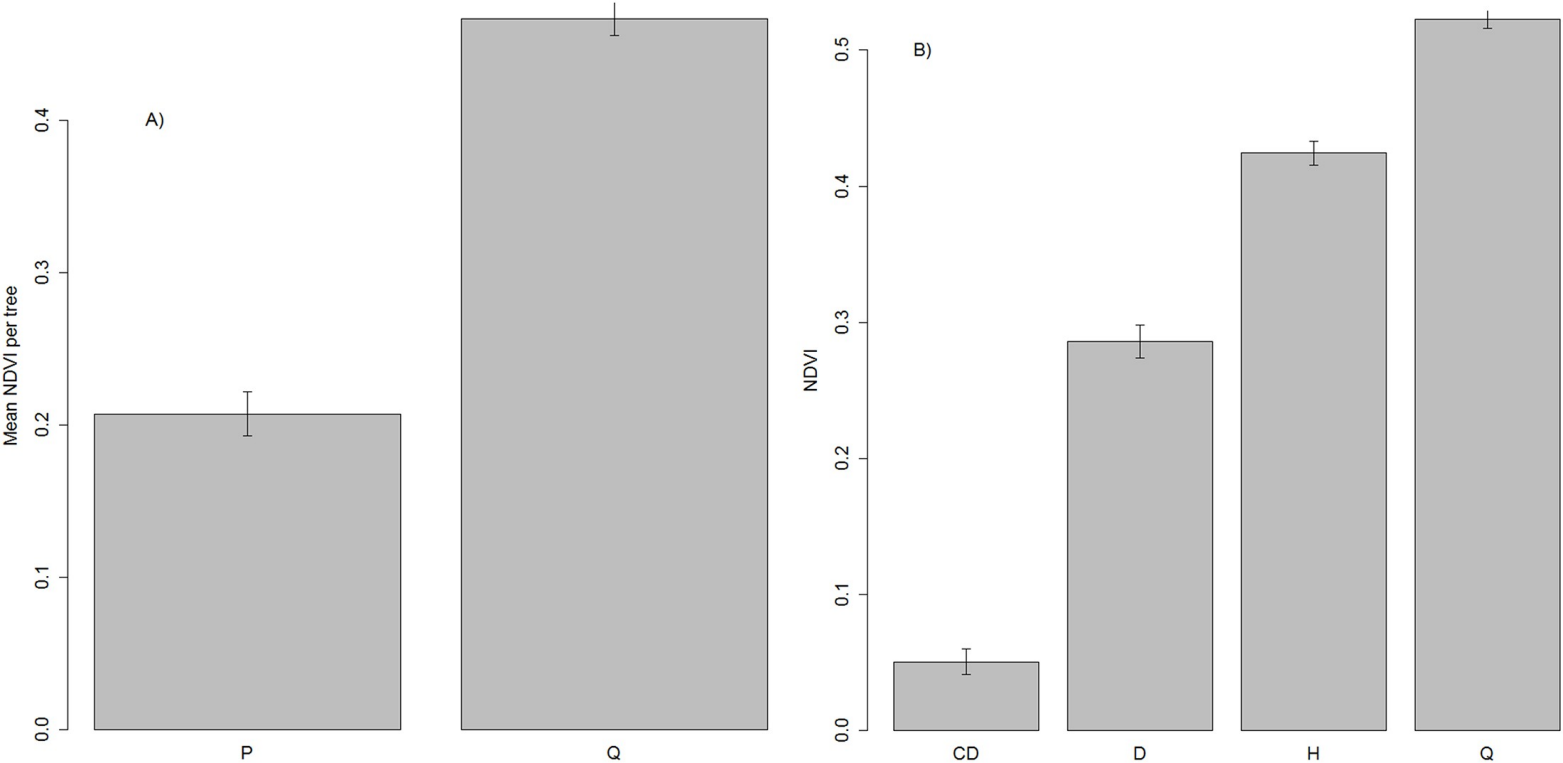
Mean NDVI at the tree level. (A) Mean NDVI values and standard error for pines (P) and holm oaks (Q). (B) completely defoliated (CD), partially defoliated (D) and non-defoliated pines (H) and holm oaks (Q) of the 110 randomly selected trees.

The NDVI-based tree species identification after identifying and delineating tree crowns was effective. We found significant differences in the mean NDVI of pines and holm oaks (p-value < 0.001; Fig 5A) and among non-defoliated, partially defoliated and completely defoliated pines and holm oaks at the same statistical significance (Fig 5B). All pines were correctly classified by the method. However, 5 of 25 holm oaks were wrongly identified, being classified as healthy pines (Tables 1 and 2). Therefore, the overall rate of success to identify the tree species was 95.5%.

**Table 1:** Confusion matrix for non-defoliated, partially defoliated and completely defoliated trees measured in the field or classified by using the multispectral imagery with the automatic ITDe.

			Multispectral imagery with automatic ITDe				
**Field**		Completely defoliated	Partially Defoliated	Non-defoliated	Holm oak	Total	Accuracy (%)
	Completely defoliated	32	11	0	0	43	74
	Partially Defoliated	0	28	1	0	29	97
	Non-defoliated	0	4	9	0	13	69
	Holm oak	0	2	3	20	25	80
	Total	32	45	13	20	110	
	Accuracy (%)	100	62	69	100		81

**Table 2:** Confusion matrix for non-defoliated, partially defoliated and completely defoliated trees measured in the field or classified by using the multispectral imagery with the manual onscreen-digitized tree segmentation (MCDe).

			Multispectral imagery with MCDe				
**Field**		Completely defoliated	Partially Defoliated	Non-defoliated	Holm oak	Total	Accuracy (%)
	Completely defoliated	40	3	0	0	43	93
	Partially Defoliated	2	24	3	0	29	83
	Non-defoliated	0	1	12	0	13	92
	Holm oak	0	1	4	20	25	80
	Total	42	29	19	20	110	
	Accuracy (%)	95	83	63	100		87

### 3.2 PPM defoliation at pixel level

We assessed the classification accuracy of the three defoliation classes (defoliated, non-defoliated, and shaded) by randomly selecting 100 pixels and validating with the reference of the orthomosaic image. The results were presented in a confusion matrix (Table 3). Out of the selected 100 pixels, 41 pixels were classified as defoliated with the NDVI threshold (NDVI = 0.27) while 34 were correctly classified as defoliated. Among 43 pixels classified as non-defoliated, 37 of them were correct (86%). Class ‘shaded’ found 10 out of 16 pixels correctly classified (62.5%). It should be noted that the number of pixels classified as defoliated was underestimated (omission errors) whereas the one classified as non-defoliated was overestimated (commission errors). The overall accuracy of classification was calculated as follows:

$$Overall\;accuracy\;(\%)=\frac{(34+37+10)\;pixels\;correctly\;classified}{100\;pixels\;in\;total}=81\%$$ (7)

Finally, the kappa coefficient indicates a level of agreement of 69%.

**Table 3:** Confusion matrix for classification assessment at the pixel level.

	Classified Image						
**Reference Image**	Class Value	Defoliated	Non-defoliated	Shaded	Total	Accuracy (%)	Kappa
	Defoliated	34	4	6	44	77	
	Non-defoliated	3	37	0	40	93	
	Shaded	4	2	10	16	63	
	Total	41	43	16	100		
	Accuracy (%)	83	86	63		81	
	Kappa						69

The classified image was based on vegetation indices while RGB orthomosaic was used as the reference image.

### 3.3 PPM defoliation at tree level

The results of our method using UAS multispectral imagery and ITDe to classify trees as non-defoliated, partially defoliated and completely defoliated pines and holm oaks was related to the data of 110 randomly selected trees though the field survey in a confusion matrix to calculate accuracy (Table 1 and Fig 4). The overall accuracy of the classification was 81.8% (94.1% when combining partially defoliated and completely defoliated trees in the same category without considering holm oaks). The method correctly distinguished all pines while 5 of the 25 holm oaks were wrongly classified as pines (Tables 1 and 2). All completely defoliated trees identified by this method were correctly classified. Among the 13 non-defoliated pines, 9 trees were correctly classified while 4 were wrongly classified as partially defoliated and none was classified as completely defoliated (Table 1). Although the classification accuracy for completely defoliated trees was 74% (Table 1), 11 completely defoliated trees in the field were classified as partially defoliated. The average percentage of defoliation of those trees was 74.8% and none of them was classified as non-defoliated.

The overall accuracy of classification with MCDe increased up to 87.2% of the 110 selected trees (95.2% when considering only non-defoliated and partially defoliated pines). Table 2 shows the accuracy for classifying the trees as non-defoliated, partially defoliated and completely defoliated pines or holm oaks by using the multispectral imagery with MCDe, which was overall higher than that with ITDe.

**Fig 6:**
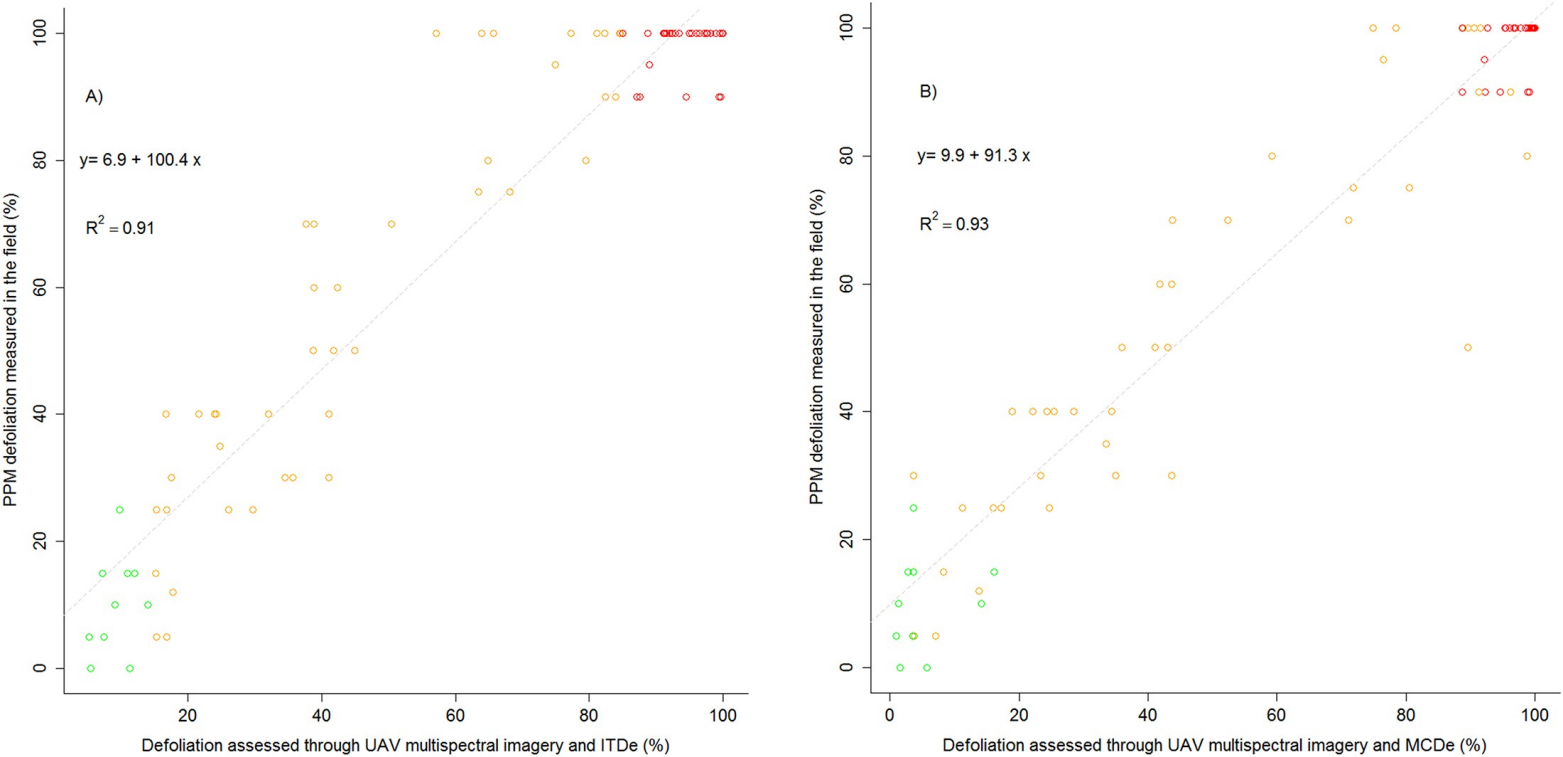
Linear regression model between PPM defoliation measured in the field and through UAS multispectral images in Codo forest site with ITDe (A) and MCDe (B). Green: non-defoliated trees; orange: partially defoliated; red: completely defoliated.

A linear regression model indicated the significant relationship between the percentage of defoliation measured through UAS multispectral images with ITDe or MCDe in pines and percentage ofdefoliation measured in the field (Fig 6). Our results showed that the relationship between the field measured defoliation and through UAS multispectral images with ITDe or MCDe was highly significant (p-value<0.001), and that the model’s predictive accuracy was very high in both cases (R^2^ = 0.91 for ITDe and R^2^ = 0.93 for MCDe).

## 4. Discussion

Under a global change context with more frequent extreme climatic events [9,55–57] PPM outbreaks are expected to become more frequent on Mediterranean coniferous and mixed forests. In this context, effective monitoring techniques are urgently required over a large spatial scales. The use of UAS-based image acquisition technology is emerging research in assessment of forest pests such as the PPM over representative spatial and temporal scales. Furthermore, the acquisition of data through UAS may also be useful to complement, or even substitute field assessments with large scale quantitative evaluations when detailed results are required. Several authors have suggested low-cost image acquisition with UAS platforms as an alternative option to assess the percentage of defoliated trees and the level of defoliation in each tree quantitatively, and obtain promising validation results with field measurements [5,27]. In this study, we have used RGB and NIR imagery to account for tree species and degree of defoliation in mix pine-oak stands. We identified pine and holm oak and classified pines as non-defoliated, partially defoliated and completely defoliated, after being identified and delineated by a local maximum algorithm on a CHM, as well as assessing the percentage of defoliation of each tree.

Our results showed that the use of UAS multispectral images through NDVI after automatically delineating tree crowns can be very useful for identifying tree species in mixed forests in order to assess PPM impacts on forests. The rate of success identifying the two tree species was very high on the 110 selected trees although further research would be needed to have a more robust methodology to be applied in other locations. In this sense, the combination of various metrics using the multispectral images, as well Green Normalized Difference Vegetation Index (GNDVI), Normalized Green-Red Vegetation Index (GRVI), Normalized Green-Blue Index (GBNDVI) or Normalized NIR-Blue Index (BNDVI), could improve the results [45,58]. Also, other recent studies used a larger sample size for species identification [44,45,58], determining when is the best time window to achieve an optimal species discrimination, an approach that could be useful given the PPM life cycle.

Field validation showed that defoliation assessment through UAS multispectral images with ITDe was accurate. The statistical models showed a significant correlation between the defoliation degree measured in the field and UAS technology at tree level and, therefore, our methodology may be used by forest managers to quantitatively assess the level of defoliation of individual trees. Moreover, this approach enabled us to classify pines among non-defoliated, partially defoliated and completely defoliated automatically after tree species identification with a classification accuracy of 81.8% with ITDe and even higher with MCDe (87.2%). This result was slightly more accurate than that obtained in the previous study [5] using RGB imagery in pure pine forest, in which the overall accuracy of the methodology to classify trees as partially defoliated or non-defoliated with MCDe was 79%. Our methodology using NDVI and removing shaded pixels detected by the ExG increased the classification accuracy between non-defoliated and partially defoliated trees up to 95.2% with MCDe. Therefore, in addition to RGB imagery, the use of multispectral imagery and vegetation indices such as NDVI may have improved assessment of PPM defoliation [27]. Although NDVI is the most widely used and tested index for monitoring of forest insect defoliation [23], further research would be needed to evaluate whether other indices among the spectral band scan improve the accuracy of PPM defoliation assessments. Furthermore, improvements in algorithms to identify and delineate individual trees may enhance the accuracy of the defoliation estimations at tree level as our results with MCDe demonstrated. In the Codo case study, the algorithm based on the UAS-derived CHM was able to successfully identify all the selected trees. Previously, other studies with different tree species, canopy cover and tree heights have found the accuracy higher than 85% in detecting individual trees, suggesting that the tree identification accuracy was high in general [41,59,60]. Yet, there is no standardized accuracy assessment procedure for ITDe, therefore, it is extremely difficult to compare ITDe algorithms unless multiple approaches are tested on a single study area using the same datasets and metrics [61]. Nonetheless, it seems evident that improvements in ITDe will enhance the estimation accuracy of PPM defoliation [62]. Our approach provides accurate PPM impact assessments with an efficient data processing to delineate individual trees in terms of time and staff, allowing the quantitative estimation of defoliation at tree-level scale in larger scales than MCDe. As expected, the accuracy levels of this methodology to analyze PPM defoliation at high resolution were higher than other previous studies using other techniques and methods with airborne laser scanning or satellite data such as Landsat or MODIS at medium resolution [5,43,63–65] and can be used in mixed forest to assess PPM defoliation at tree level.

At the pixel level, the overall classification accuracy among partially defoliated, non-defoliated and shaded was 81% in the high resolution NIR imagery, which was as high as that at the tree level with ITDe. The use of NDVI in general has the advantage of detecting non-defoliated pixels that cannot be visibly detected by RGB imagery due to shade as has been demonstrated in agricultural areas [53], but we think that it is still necessary to achieve some improvements in the multispectral sensors and/or in radiometric calibrations for forest areas as Codo. This type of invisibility in the orthomosaic image may make it difficult to validate the classification accuracy of predicted non-defoliated pixels based on NDVI. Another limitation was the one hour difference between the flights with the RGB camera and the multispectral camera. This temporal difference may have contributed to slight increases in the uncertainty in shaded pixels. In the future study the simultaneous flight with both RGB and multispectral cameras, as the UAS technology advances, may improve our methodology and the results.

The use of UAS technology offers several advantages when applied to the defoliation assessment caused by insect pests in spite of the fact that substantial investment in equipment, infrastructure and training of people is necessary. The key highlights of those advantages include (1) data acquisition is usually more efficient in terms of time, quality and manpower, (2) the technology has a great capacity to monitor and assess areas with difficult access (3) researchers can easily derive quantitative methods to estimate defoliation at tree level and (4) this information can be analyzed at forest stand scale which is the work scale of forest managers and eventually be repeated in time thus providing the potential for the development of long term forest health monitoring programs. Finally, (5) UAS high-resolution data can be a great source of information to calibrate medium resolution remote sensing information derived from satellites to map information in coarser scales (Fraser et al, 2017; Pla et al, 2017). Nevertheless, it should be noted that several methodological constraints need to be considered when planning for the large area deployment of UAS technologies in the estimation of defoliation levels such as the regulations to use UAS in urban areas and areas located near airfields.

## 5. Conclusions

In this study, we investigated the use of multispectral high-resolution imagery acquired from a UAS platform and image processing techniques to quantitatively assess PPM impact on a pine-oak mixed forest at tree level. Overall, this research suggested that UAS imagery and its derived products, such as canopy height model and normalized difference vegetation index, enabled us to estimate tree species, count individual trees with acceptable accuracy and assess defoliation using canopy cover at tree level by classifying pines non-defoliated, partially defoliated and completely defoliated automatically with high accuracy. Moreover, the accuracy of our proposed methodology at tree level was higher than previous studies. This proposed framework highlights the future potential of UAS, multispectral imagery and structure-from-motion algorithms for individual tree detection, PPM quantification, qualification and monitoring. Thus, we believe that the promising results presented here in should inspire further research and applications to the forest health assessments.
